# Cross-National Analysis of the Associations between Traumatic Events and Suicidal Behavior: Findings from the WHO World Mental Health Surveys

**DOI:** 10.1371/journal.pone.0010574

**Published:** 2010-05-13

**Authors:** Dan J. Stein, Wai Tat Chiu, Irving Hwang, Ronald C. Kessler, Nancy Sampson, Jordi Alonso, Guilherme Borges, Evelyn Bromet, Ronny Bruffaerts, Giovanni de Girolamo, Silvia Florescu, Oye Gureje, Yanling He, Viviane Kovess-Masfety, Daphna Levinson, Herbert Matschinger, Zeina Mneimneh, Yosikazu Nakamura, Johan Ormel, Jose Posada-Villa, Rajesh Sagar, Kate M. Scott, Toma Tomov, Maria Carmen Viana, David R. Williams, Matthew K. Nock

**Affiliations:** 1 Department of Psychiatry, Groote Schuur Hospital, Cape Town, South Africa; 2 Department of Health Care Policy, Harvard Medical School, Boston, Massachusetts, United States of America; 3 Health Services Research Unit, Institut Municipal d'Investigació Mèdica (IMIM-Hospital del Mar), CIBER en Epidemiología y Salud Pública (CIBERESP), Barcelona, Spain; 4 Department of Epidemiological Research, Division of Epidemiological and Psychosocial Research, National Institute of Psychiatry (Mexico) and Metropolitan Autonomous University, Mexico City, Mexico; 5 Department of Psychiatry, State University of New York at Stony Brook, New York, United States of America; 6 University Hospital Gasthuisberg, Leuven, Belgium; 7 Instituto di Ricovero e Cura a Carattere Scientifico (IRCCS), Centro San Giovanni di Dio Fatebenefratelli, Brescia, Italy; 8 National School of Public Health and Health Services Management, Bucharest, Romania; 9 University College Hospital, Ibadan, Nigeria; 10 Shanghai Mental Health Center, Shanghai, China; 11 EA 4069 Université Paris Descartes, Paris, France; 12 Research and Planning, Mental Health Services, Ministry of Health, Jerusalem, Israel; 13 Clinic of Psychiatry, University of Leipzig, Leipzig, Germany; 14 Institute for Development, Research, Advocacy and Applied Care (IDRAAC), Beirut, Lebanon; 15 Survey Research Center, Institute for Social Research, University of Michigan, Ann Arbor, Michigan, United States of America; 16 School of Public Health, Jichi Medical University, Tochigi-ken, Japan; 17 Department of Psychiatry and Psychiatric Epidemiology, University Medical Center Groningen, University Center for Psychiatry, Groningen, Netherlands; 18 Centro Medico de la Sabana, Universidad Javerina, Bogota, Colombia; 19 Department of Psychiatry, All India Institute of Medical Sciences, Delhi, India; 20 Department of Psychological Medicine, Wellington School of Medicine and Health Sciences, Otago, New Zealand; 21 Institute for Human Relations, New Bulgarian University, Sofia, Bulgaria; 22 Department of Psychiatric Epidemiology, Institute of Psychiatry, School of Medicine, University of São Paulo, São Paulo, Brazil; 23 Department of Society, Human Development, and Health, Harvard School of Public Health, Boston, Massachusetts, United States of America; 24 Department of Psychology, Harvard University, Cambridge, Massachusetts, United States of America; CIET, Canada

## Abstract

**Background:**

Community and clinical data have suggested there is an association between trauma exposure and suicidal behavior (i.e., suicide ideation, plans and attempts). However, few studies have assessed which traumas are uniquely predictive of: the first onset of suicidal behavior, the progression from suicide ideation to plans and attempts, or the persistence of each form of suicidal behavior over time. Moreover, few data are available on such associations in developing countries. The current study addresses each of these issues.

**Methodology/Principal Findings:**

Data on trauma exposure and subsequent first onset of suicidal behavior were collected via structured interviews conducted in the households of 102,245 (age 18+) respondents from 21 countries participating in the WHO World Mental Health Surveys. Bivariate and multivariate survival models tested the relationship between the type and number of traumatic events and subsequent suicidal behavior. A range of traumatic events are associated with suicidal behavior, with sexual and interpersonal violence consistently showing the strongest effects. There is a dose-response relationship between the number of traumatic events and suicide ideation/attempt; however, there is decay in the strength of the association with more events. Although a range of traumatic events are associated with the onset of suicide ideation, fewer events predict which people with suicide ideation progress to suicide plan and attempt, or the persistence of suicidal behavior over time. Associations generally are consistent across high-, middle-, and low-income countries.

**Conclusions/Significance:**

This study provides more detailed information than previously available on the relationship between traumatic events and suicidal behavior and indicates that this association is fairly consistent across developed and developing countries. These data reinforce the importance of psychological trauma as a major public health problem, and highlight the significance of screening for the presence and accumulation of traumatic exposures as a risk factor for suicide ideation and attempt.

## Introduction

Suicidal behavior (i.e, suicide ideation, plans, or attempts) is an important public health problem that results in significant morbidity and mortality and is a major contributor to the global burden of disease [1], [2]. Although most suicide attempts do not result in death, such attempts carry a risk for serious injury, are associated with suffering, and increase the risk for subsequent attempts [3]–[5]. There is an urgent need for research to better understand risk factors for suicidal behavior [6]–[8]. Psychiatric disorders are among the strongest predictors of suicidal behavior [9], [10]; however, recent data from the World Mental Health Surveys indicate that 31–57% of suicide attempts are not associated with prior psychiatric disorder [11], highlighting the need to understand what other factors might increase the risk of suicidal behavior. There is growing interest in understanding the environmental and genetic influences on suicidal behavior [12], and recent evidence indicates that environmental factors have a stronger influence on the occurrence of negative psychological outcomes (e.g., depression, suicidal behavior) than do known genetic factors [13]. A particularly important potential environmental contributor to suicidal behavior may be exposure to psychological trauma.

Several studies have reported an association between early childhood abuse and subsequent suicidal behavior [14]–[16]. However, other recent data suggest that exposure to psychological trauma (whether assaultive or non-assaultive) is not an independent predictor of subsequent suicide attempts outside the context of post traumatic stress disorder [PTSD; 17]. Several additional questions remain about the nature of the putative association between exposure to trauma events and suicidality.

First, few studies have assessed which traumas are uniquely predictive of suicidal behavior and its persistence. Traumas often occur in contexts characterized by significant social disruption, particularly among subjects with early adversity. Multivariate analyses, controlling for the effects of different traumatic events may, however, be able to show that certain traumas have a particularly high association with suicidality. For instance, witnessing violent events is strongly associated with being the victim of a violent event, and it would be useful to test the unique association between each type of event and suicidal behavior. Moreover, it is possible that certain types of events, such as those in which the person is physically assaulted or sexually abused, are more distressing and more strongly associated with subsequent suicidal behavior than non-violent events. However, such distinctions have not been carefully tested in prior research—as very large samples are needed to test these more fine-grained associations between specific types of traumatic events and suicidal behavior.

Second, there are few data on the extent to which traumatic events predict the progression from suicide ideation to plans and attempts. Although exposure to traumatic events may be predictive of suicide ideation, it may not necessarily be useful in predicting which people with suicide ideation go on to make suicide plans and attempts. Recent research has shown that many known risk factors for suicidal behavior such as, the presence of a depressive disorder, predict the onset of suicide ideation, but not which people with ideation go on to make a suicide attempt [11]. Despite its potential clinical importance, this issue has not been well studied. Similarly, virtually no studies have examined predictors of the persistence of suicidal behavior over time (i.e., number of years from the first onset to the most recent occurrence of suicidal behavior). Such information is important for understanding the nature of suicidal behavior and for the purposes of clinical monitoring and risk assessment.

Third, most studies on the association of trauma and suicidality to date have been undertaken in developed, high-income countries. There may be different associations between trauma and suicidality in developing countries, where traumatic events may be more prevalent and of different types than those experienced in developed countries [18]. Indeed, recent work has suggested that PTSD is a stronger predictor of suicide attempts in developing countries (odds ratio = 5.6) than in developed countries (odds ratio = 3.0) [11], which may be reflective of such differences. Accurate information on the risk factors for suicidal behavior in both developed and developing countries is needed for the creation of better screening, prevention, and intervention programs around the globe.

The current study uses data from the WHO World Mental Health Surveys to address each of these issues. This series of coordinated epidemiological surveys was carried out in a broad range of countries, and included a detailed assessment of exposure to psychological traumas, as well as a comprehensive survey of suicidal behavior (i.e., suicide ideation, plans, and attempts) [19]. The aims of the study were to examine the unique associations between psychological trauma and suicidal behavior, and to consider the effects of such trauma on multiple forms of suicidality, in high-, middle-, and low-income countries.

## Methods

### Respondent samples

The WMH surveys were carried out in 21 countries in: Africa (Nigeria; South Africa), the Americas (Brazil; Colombia; Mexico; United States), Asia and the Pacific (India; Japan; New Zealand; Beijing and Shanghai in the People's Republic of China), Europe (Belgium; Bulgaria; France; Germany; Italy; the Netherlands; Romania; Spain; Ukraine), and the Middle East (Israel; Lebanon). The World Bank [20] classifies Colombia, India, Nigeria, China, and Ukraine as low and lower-middle income countries (hereafter “low income countries”); Brazil, Bulgaria, Lebanon, Mexico, Romania, and South Africa as upper-middle income countries (“middle income countries”); and all other survey countries as high income countries. Respondents were selected in most WMH countries using a stratified multistage clustered-area probability sampling strategy. The total sample size was 102,245 (age 18+), with individual country sample sizes ranging from 2,357 in Romania to 12,790 in New Zealand. The weighted average response rate across all countries was 71.9% (Table 1).

**Table 1:** WMH sample characteristics by World Bank income categories^1^.

Country by income category	Survey^1^ ^2^	Sample Characteristics^3^	Field Dates	Age Range	Sample Size			Response Rate^5^
					Part I	Part II	Part II and Age≤44^4^	
**Low and Lower-middle**								
Colombia	NSMH	Stratified multistage clustered area probability sample of household residents in all urban areas of the country (approximately 73% of the total national population)	2003	18–65	4426	2381	1731	87.7
India	WMHI	Stratified multistage clustered area probability sample of household residents in Pondicherry region. NR	2003–5	18+	2992	1373	642	98.6
Nigeria	NSMHW	Stratified multistage clustered area probability sample of households in 21 of the 36 states in the country, representing 57% of the national population. The surveys were conducted in Yoruba, Igbo, Hausa and Efik languages.	2002–3	18+	6752	2143	1203	79.3
PRC	B-WMHS-WMH	Stratified multistage clustered area probability sample of household residents in the Beijing and Shanghai metropolitan areas.	2002–3	18+	5201	1628	570	74.7
Ukraine	CMDPSD	Stratified multistage clustered area probability sample of household residents. NR	2002	18+	4725	1720	541	78.3
Total					24096	9245	4687	
**Upper-middle**								
Brazil	São Paulo Megacity	Stratified multistage clustered area probability sample of household residents in the São Paulo metropolitan area.	2005–7	18+	5037	2942	—	81.3
Bulgaria	NSHS	Stratified multistage clustered area probability sample of household residents. NR	2003–7	18+	5318	2233	741	72.0
Lebanon	LEBANON	Stratified multistage clustered area probability sample of household residents. NR	2002–3	18+	2857	1031	595	70.0
Mexico	M-NCS	Stratified multistage clustered area probability sample of household residents in all urban areas of the country (approximately 75% of the total national population).	2001–2	18–65	5782	2362	1736	76.6
Romania	RMHS	Stratified multistage clustered area probability sample of household residents. NR	2005–6	18+	2357	2357	—	70.9
South Africa	SASH	Stratified multistage clustered area probability sample of household residents. NR	2003–4	18+	4315	4315	—	87.1
Total					25666	15240	3072	
**High**								
Belgium	ESEMeD	Stratified multistage clustered probability sample of individuals residing in households from the national register of Belgium residents. NR	2001–2	18+	2419	1043	486	50.6
France	ESEMeD	Stratified multistage clustered sample of working telephone numbers merged with a reverse directory (for listed numbers). Initial recruitment was by telephone, with supplemental in-person recruitment in households with listed numbers. NR	2001–2	18+	2894	1436	727	45.9
Germany	ESEMeD	Stratified multistage clustered probability sample of individuals from community resident registries. NR	2002–3	18+	3555	1323	621	57.8
Israel	NHS	Stratified multistage clustered area probability sample of individuals from a national resident register. NR	2002–4	21+	4859	4859	—	72.6
Italy	ESEMeD	Stratified multistage clustered probability sample of individuals from municipality resident registries. NR	2001–2	18+	4712	1779	853	71.3
Japan	WMHJ2002–2006	Un-clustered two-stage probability sample of individuals residing in households in eleven metropolitan areas	2002–6	20+	4129	1682	547	55.1
Netherlands	ESEMeD	Stratified multistage clustered probability sample of individuals residing in households that are listed in municipal postal registries. NR	2002–3	18+	2372	1094	516	56.4
New Zealand^6^	NZMHS	Stratified multistage clustered area probability sample of household residents. NR	2004–5	18+	12790	7312	4119	73.3
Spain	ESEMeD	Stratified multistage clustered area probability sample of household residents. NR	2001–2	18+	5473	2121	960	78.6
United States	NCS-R	Stratified multistage clustered area probability sample of household residents. NR	2002–3	18+	9282	5692	3197	70.9
Total					52485	28341	12026	

1 The World Bank. (2008). Data and Statistics. Accessed May 12, 2009 at: http://go.worldbank.org/D7SN0B8YU0.

2 NSMH (The Colombian National Study of Mental Health); WMHI (World Mental Health India); NSMHW (The Nigerian Survey of Mental Health and Wellbeing); B-WMH (The Beijing World Mental Health Survey); S-WMH (The Shanghai World Mental Health Survey); CMDPSD (Comorbid Mental Disorders during Periods of Social Disruption); NSHS (Bulgaria National Survey of Health and Stress); LEBANON (Lebanese Evaluation of the Burden of Ailments and Needs of the Nation); M-NCS (The Mexico National Comorbidity Survey); RMHS (Romania Mental Health Survey); SASH (South Africa Health Survey); ESEMeD (The European Study Of The Epidemiology Of Mental Disorders); NHS (Israel National Health Survey); WMHJ2002–2006 (World Mental Health Japan Survey); NZMHS (New Zealand Mental Health Survey); NCS-R (The US National Comorbidity Survey Replication).

3 Most WMH surveys are based on stratified multistage clustered area probability household samples in which samples of areas equivalent to counties or municipalities in the US were selected in the first stage followed by one or more subsequent stages of geographic sampling (e.g., towns within counties, blocks within towns, households within blocks) to arrive at a sample of households, in each of which a listing of household members was created and one or two people were selected from this listing to be interviewed. No substitution was allowed when the originally sampled household resident could not be interviewed. These household samples were selected from Census area data in all countries other than France (where telephone directories were used to select households) and the Netherlands (where postal registries were used to select households). Several WMH surveys (Belgium, Germany, Italy) used municipal resident registries to select respondents without listing households. The Japanese sample is the only totally un-clustered sample, with households randomly selected in each of the four sample areas and one random respondent selected in each sample household. 17 of the 20 surveys are based on nationally representative (NR) household samples, while two others are based on nationally representative household samples in urbanized areas (Colombia, Mexico).

4 Brazil, Israel, Romania, and South Africa did not have an age restricted Part II sample. All other countries, with the exception of India, Nigeria, People's Republic of China, and Ukraine (which were age restricted to≤39) were age restricted to≤44.

5 The response rate is calculated as the ratio of the number of households in which an interview was completed to the number of households originally sampled, excluding from the denominator households known not to be eligible either because of being vacant at the time of initial contact or because the residents were unable to speak the designated languages of the survey. The weighted average response rate is 71.9%.

6 New Zealand interviewed respondents 16+ but for the purposes of cross-national comparisons we limit the sample to those 18+.

### Procedures

All surveys were conducted face-to-face by trained lay interviewers. Standardized interviewer training procedures, WHO translation protocols for all study materials, and quality control procedures for interviewer and data accuracy that have been consistently employed across all WMH countries are described in more detail elsewhere [21], [22]. All respondents completed a Part I interview that contained core diagnostic assessments, including the assessment of suicidal behavior (except in Israel, Romania, and South Africa where all respondents completed both Part I and Part II). All Part I respondents who met criteria for any disorder and a sub-sample of approximately 25% of the rest of the respondents were administered a Part II interview that assessed potential correlates and disorders of secondary interest (*n* = 52,824, age 18+). Data were weighted to adjust for this differential sampling of Part II respondents, to adjust for differential probabilities of selection within households, and to match samples to population socio-demographic distributions. Informed consent was obtained before beginning interviews in all countries.

#### Ethics Statement

Procedures for obtaining informed consent and protecting human subjects were approved and monitored for compliance by the Institutional Review Boards of organizations coordinating surveys in each country based on a template developed by the WMH Data Collection Coordinating Centre. A complete list of the participating IRBs, type of consent obtained, procedures for documenting consent, and incentives offered for participation is available at: http://www.hcp.med.harvard.edu/wmh/ftpdir/nationalsample_Ethics_statement.pdf.

### Measures

#### Traumatic events

Traumatic events were assessed using the WMH version of the WHO Composite International Diagnostic Interview (CIDI) Version 3.0, a fully structured diagnostic interview administered by trained lay interviewers [21], which includes a screen for traumatic events as part of the module for the diagnosis of PTSD. The traumatic events assessed in this module incorporate those from various categories, including: (1) natural and man-made disasters and accidents; (2) combat, war, and refugee experiences; (3) sexual and interpersonal violence; (4) witnessing or perpetrating violence; and (5) death or trauma to a loved one. Each type of event was queried separately. For instance, if a person experienced a natural disaster during which a loved one was killed, they could endorse the experience of both traumatic events. This allowed for an examination of the independent effects of each type of event. Respondent age at the time of occurrence of each event was recorded and traumatic events were treated as time varying covariates in each statistical model except for persistence models, for which traumatic events were observed at the time of each suicide outcome and treated as a constant throughout the respondent's life course. Only traumatic events that occurred temporally prior to each suicidal behavior being examined were tested as predictors in each model.

#### Suicidal behavior

Suicidal behavior was assessed using the Suicidality Module of the WMH-CIDI [21]. This module includes an assessment of the lifetime occurrence, age-of-onset, and age of most recent episode of suicide ideation (“Have you ever seriously thought about committing suicide?”), plans (“Have you ever made a plan for committing suicide?”), and attempts (“Have you ever attempted suicide?”). Consistent with our goal of examining relationships of mental disorders with a continuum of suicidal behaviors, we considered five dated lifetime history outcomes in a series of nested survival analyses (see below for analysis methods): (1) suicide ideation in the total sample, (2) suicide attempt in the total sample, (3) suicide plan among ideators; (4) suicide attempt among ideators with a plan (‘planned attempt’); and (5) suicide attempt among ideators without a plan (‘unplanned attempt’).

### Analysis methods

We examined the associations among temporally prior traumatic events (i.e., time-varying covariates) and subsequent suicidal behaviors using discrete-time survival models with person-year as the unit of analysis [23]. Controls for all models include person-year, country, demographic factors (age, gender, time-varying education, time-varying marriage), interactions between life course (3 dichotomous dummies representing early, middle, and later years in the person's life) and demographic factors, parent psychopathology [24], and childhood adversities [15] (additional details available upon request). Missing values for control variables were estimated using multiple imputation [25]. We estimated survival models that were bivariate (i.e., including only one traumatic event at a time) as well as multivariate (i.e., including all traumatic events simultaneously) in predicting each of the five suicide outcomes. Two types of multivariate models were tested: One including all types of traumatic events simultaneously (multivariate additive), and one including both the type and number of traumatic events experienced by each respondent as dummy variables (multivariate interactive). We also tested the associations between traumatic events and the persistence of suicidal behavior using backward recurrence models [26]–[28]. Such models use a person-year survival approach; however, instead of predicting a future event, we predicted the most recent episode of the event of interest (e.g., most recent suicide attempt) among those who had ever had an initial event (e.g., first suicide attempt) looking backwards in time from the year of interview. For example, a person who made a suicide attempt for the first time at age 25, for the last time at age 30, and who is currently 32 years-old would have three years in their data file coded: 1 (year 30) and 0, 0 (years 31 and 32). A person who made a suicide attempt for the first time at age 25, never made another attempt, and currently is 32 years-old would have 7 time-since-onset (TSO) person-years in their data file all coded 0. In these models age of onset (AOO) and TSO are statistically controlled and so the models provide an indirect estimate of the persistence of each outcome of interest. Studies comparing the results from backward recurrence models with prospective time-to-next-event survival models indicate that the former provide generally good approximations of the coefficients obtained in the latter [29]. Finally, we calculated population attributable risk proportions (PARPs) to examine the population-level effects of traumatic events on suicidal behavior. PARPs represent the proportion of observed cases of the outcome that would be prevented if specific predictor variables could be eliminated, based on the assumption that the ORs in the model accurately represent causal effects of the predictors.

In all analyses, coefficients and standard errors were exponentiated for ease of interpretation and are reported as odds ratios (ORs) with 95% confidence intervals (CIs). Standard errors were estimated with the Taylor series method [30] using SUDAAN software [31] to adjust for weighting and clustering. Multivariate significance was evaluated with Wald χ^2^ tests based on design-corrected coefficient variance–covariance matrices. In each analysis, associations between traumatic events and suicide outcomes were adjusted for the possible influence of country differences, sex, age, educational attainment, marriage, parental psychopathology, and childhood adversities. All significance tests were evaluated using .05-level two-sided tests. Given the large sample size and multiple analyses conducted in this study, we focus on the magnitude of observed effects rather than on statistical significance in interpreting the importance of study results.

## Results

### Traumatic events

Traumatic events were fairly common across each sample, occurring among 2.1–30.5% of respondents in each country. The most commonly reported trauma was the death of a loved one (30.5%), followed by witnessing violence (21.8%). More than 10% of the respondents reported interpersonal violence (18.8%), accidents (17.7%), exposure to war (16.2%), or trauma to a loved one (12.5%). Other traumas were less common and all under the 10% level.

In the pooled sample, lifetime suicide ideation and attempts were reported by 9.6% (or *n* = 8,126) and 2.8% (or *n* = 2,778) of respondents, respectively. Among ideators, 34.8% (or *n* = 3,252) developed a suicide plan, and 55.7% of these respondents (or *n* = 1,871) made a suicide attempt. Among the ideators (*n* = 8.126), 65.2% (or *n* = 4,874) did not make a suicide plan, and, of those without a plan, 15.3% (or *n* = 907) made an attempt.

Among respondents with a history of suicide attempt, almost one in five (20.9%) reported loss of a loved one, and about one in six (16.0%) reported interpersonal violence. Traumas ranged, however, from 1.2% to 20.9%, and we found roughly comparable patterns for estimates of traumas in the other suicide-related behaviors included. More detailed results reported for each adversity and each type of suicidal behavior after disaggregating for income categories are available upon request.

### Bivariate associations of traumatic events with lifetime suicidal behavior

Tabulation of bivariate associations (Table 2) shows that the majority of traumatic events are significantly associated with lifetime suicide ideation and suicide attempt. The ORs are highest for sexual (ORs = 2.2–2.6 [95% confidence interval: 2.0–3.1]) and interpersonal (ORs = 1.8–1.9 [CI: 1.6–2.2]) violence. Among those with suicide ideation, traumas generally are not predictive of suicide plan, planned attempt, or unplanned attempt. A similar pattern of findings holds in high-, middle-, and low-income countries (data available upon request). However, in the cross-national sample, among those with suicide ideation, natural disaster is positively associated with suicide plan (OR = 1.3 [CI: 1.1–1.6]), exposure to war is positively associated with planned attempt (OR = 1.6 [CI: 1.0–2.5]), and sexual violence is positively associated with unplanned attempt (OR = 1.5 [CI: 1.1–2.0]).

**Table 2:** Bivariate model for associations between traumatic events and suicidal behavior^1^.

	Among Total Sample		Plan among ideators	Attempt among ideators with a plan	Attempt among ideators without a plan
	Ideation	Attempt			
Type of Traumatic Events	OR(95% CI)	OR(95% CI)	OR(95% CI)	OR(95% CI)	OR(95% CI)
Disasters/Accidents					
All Man Made Disasters	1.3* (1.1–1.5)	1.3* (1.0–1.6)	1.2 (0.9–1.5)	0.9 (0.6–1.3)	1.0 (0.7–1.4)
Natural Disaster	1.1 (0.9–1.3)	1.3 (1.0–1.6)	1.3* (1.1–1.6)	1.2 (0.8–1.7)	1.3 (0.9–2.0)
Accident	1.5* (1.4–1.7)	1.4* (1.2–1.7)	1.1 (0.9–1.3)	0.7* (0.5–1.0)	0.9 (0.7–1.2)
War/Combat/Refugee Experiences					
Exposure to War	1.1 (0.9–1.2)	1.3* (1.0–1.7)	1.1 (0.9–1.4)	1.6* (1.0–2.5)	0.7 (0.4–1.1)
Combat	1.2 (1.0–1.6)	0.9 (0.6–1.3)	1.3 (0.9–1.9)	1.2 (0.6–2.6)	0.5 (0.2–1.1)
Refugee	1.1 (0.9–1.5)	1.2 (0.8–1.8)	1.0 (0.6–1.7)	1.6 (0.8–3.2)	1.2 (0.5–3.0)
Sexual/Interpersonal Violence					
Sexual Violence	2.2* (2.0–2.4)	2.6* (2.2–3.1)	1.1 (0.9–1.3)	0.9 (0.6–1.2)	1.5* (1.1–2.0)
Interpersonal Violence	1.8* (1.6–2.0)	1.9* (1.6–2.2)	1.1 (0.9–1.3)	0.7* (0.5–0.9)	1.1 (0.8–1.5)
Witness/Perpetrator Violence					
Witness Violence	1.4* (1.2–1.5)	1.4* (1.2–1.6)	1.2 (1.0–1.4)	0.9 (0.7–1.3)	1.2 (0.9–1.6)
Perpetrator Violence	1.6* (1.3–1.9)	1.5* (1.1–2.0)	1.2 (0.9–1.7)	0.6 (0.4–1.1)	1.1 (0.6–2.0)
Loss/Trauma					
Death of Loved One	1.2* (1.1–1.3)	1.1 (1.0–1.3)	0.9 (0.8–1.0)	0.9 (0.7–1.1)	0.8 (0.6–1.0)
Trauma to Loved one	1.3* (1.1–1.4)	1.3* (1.1–1.6)	0.8* (0.7–1.0)	1.0 (0.7–1.3)	1.0 (0.7–1.3)
All Others	1.5* (1.4–1.8)	1.6* (1.3–1.9)	1.0 (0.8–1.2)	0.9 (0.7–1.3)	1.0 (0.7–1.4)

*Significant at the .05 level, two-sided test.

1 Assessed in Part II sample due to having Part II controls. Each row represents a separate bivariate model; some models do not include data from all countries if the country is missing the variable. India and Brazil were dropped in the bivariate model for Combat, Exposure to War and Refugees; and Brazil was dropped in the bivariate model for Natural Disaster. For Israel, the entire sample is coded “Yes” for exposure to war with the age of onset set to the age they moved to Israel. Controls for all models included person-year, country, demographic factors (age, sex, time-varying education, time-varying marriage), interactions between life course (3 dichotomous dummies representing early, middle, and later years in the person's life) and demographic variables, parent psychopathology, and childhood adversities (additional details available upon request).

### Multivariate associations of traumatic events with lifetime suicidal behavior

After controlling for the effects of other traumatic events, there are fewer significant associations between traumatic events and both suicide ideation and suicide attempt (Table 3). ORs remained highest for sexual violence (ORs = 2.0–2.3 [CI: 1.8–2.7]) and interpersonal violence (ORs = 1.6 [CI: 1.4–1.9]). Disaggregation of the associations between traumatic events and suicide attempts again suggests that they are largely due to traumatic events predicting suicide ideation rather than to the progression from suicide ideation to attempt. A similar pattern of findings is seen in high-, middle-, and low-income countries (data available upon request). Again, in the cross-national sample, among those with suicide ideation, natural disaster is positively associated with suicide plan (OR = 1.3 [CI: 1.0–1.6]), exposure to war is positively associated with planned attempt (OR = 1.7 [CI: 1.1–2.6]), and sexual violence is positively associated with unplanned attempt (OR = 1.5 [CI: 1.1–2.1]).

**Table 3:** Multivariate model for associations between traumatic events and suicidal behavior^1^.

	Among Total Sample		Plan among ideators	Attempt among ideators with a plan	Attempt among ideators without a plan
	Ideation	Attempt			
Type of Traumatic Events	OR(95% CI)	OR(95% CI)	OR(95% CI)	OR(95% CI)	OR(95% CI)
Disasters/Accidents					
All Man Made Disasters	1.1 (1.0–1.3)	1.1 (0.9–1.3)	1.1 (0.9–1.4)	0.9 (0.6–1.4)	1.0 (0.7–1.4)
Natural Disaster	0.9 (0.8–1.1)	1.1 (0.9–1.4)	1.3* (1.0–1.6)	1.2 (0.9–1.8)	1.4 (1.0–2.0)
Accident	1.3* (1.2–1.5)	1.2* (1.0–1.4)	1.0 (0.9–1.2)	0.7 (0.5–1.0)	0.9 (0.6–1.1)
War/Combat/Refugee Experiences					
Exposure to War	1.0 (0.8–1.1)	1.2 (0.9–1.5)	1.1 (0.8–1.4)	1.7* (1.1–2.6)	0.6 (0.4–1.1)
Combat	1.0 (0.8–1.3)	0.7* (0.5–1.0)	1.2 (0.8–1.8)	1.2 (0.5–2.7)	0.4 (0.2–1.1)
Refugee	1.0 (0.8–1.3)	1.0 (0.6–1.6)	0.9 (0.5–1.6)	1.5 (0.8–3.1)	1.5 (0.6–3.8)
Sexual/Interpersonal Violence					
Sexual Violence	2.0* (1.8–2.2)	2.3* (2.0–2.7)	1.0 (0.8–1.3)	0.9 (0.7–1.2)	1.5* (1.1–2.1)
Interpersonal Violence	1.6* (1.4–1.8)	1.6* (1.4–1.9)	1.0 (0.9–1.3)	0.7* (0.5–1.0)	1.1 (0.8–1.4)
Witness/Perpetrator Violence					
Witness Violence	1.2* (1.0–1.3)	1.2* (1.0–1.4)	1.2 (1.0–1.4)	1.0 (0.7–1.4)	1.2 (0.9–1.7)
Perpetrator Violence	1.2 (1.0–1.5)	1.2 (0.9–1.7)	1.1 (0.8–1.6)	0.7 (0.4–1.2)	1.3 (0.7–2.3)
Loss/Trauma					
Death of Loved One	1.1 (1.0–1.2)	1.0 (0.9–1.1)	0.9* (0.7–1.0)	0.9 (0.7–1.1)	0.8* (0.6–1.0)
Trauma to Loved one	1.1 (0.9–1.2)	1.1 (0.9–1.4)	0.8* (0.7–1.0)	1.0 (0.8–1.3)	1.0 (0.7–1.3)
All Others	1.3* (1.2–1.5)	1.4* (1.1–1.7)	1.0 (0.8–1.2)	1.0 (0.7–1.3)	1.0 (0.7–1.4)

*Significant at the .05 level, two-sided test.

1 Assessed in Part II sample due to having Part II controls. Some countries were missing part of the trauma variables and were coded “No” for those variables: Combat, Exposure to War, Refugee were all coded “No” for India and Brazil, and Natural Disaster also coded “No” for Brazil. For Israel, the entire sample is coded “Yes” for exposure to war with the age of onset set to the age they moved to Israel. Controls for all models included person-year, country, demographic factors (age, sex, time-varying education, time-varying marriage), interactions between life course (3 dichotomous dummies representing early, middle, and later years in the person's life) and demographic variables, parent psychopathology, and childhood adversities (additional details available upon request).

### Effects of the number of traumatic events

There is a positive relationship between the number of traumatic events experienced and the odds of subsequent suicide ideation and suicide attempt (Table 4). Once again, these associations are largely due to traumatic events predicting suicide ideation, rather than the progression from suicide ideation to suicide plan and attempt. For instance, the ORs for suicide attempt increase from 1.6 (CI: 1.4–1.9) among those with one traumatic event (relative to those with zero events) to 4.3 (CI: 2.8–6.5) among those with six traumatic events.

**Table 4:** Associations between number of traumatic events and suicidal behavior^1^.

	Among Total Sample		Plan among ideators	Attempt among ideators with a plan	Attempt among ideators without a plan
	Ideation	Attempt			
Number of Traumatic Events^2^	OR(95% CI)	OR(95% CI)	OR(95% CI)	OR(95% CI)	OR(95% CI)
1	1.5* (1.4–1.6)	1.6* (1.4–1.9)	1.0 (0.9–1.2)	0.8 (0.6–1.0)	0.9 (0.7–1.2)
2	1.9* (1.8–2.1)	2.1* (1.8–2.5)	1.1 (0.9–1.4)	0.8 (0.6–1.1)	1.1 (0.8–1.5)
3	2.1* (1.8–2.4)	2.1* (1.8–2.6)	1.0 (0.8–1.3)	0.6* (0.4–0.8)	1.0 (0.7–1.4)
4	2.2* (1.8–2.6)	2.4* (1.8–3.1)	1.0 (0.7–1.3)	0.7 (0.4–1.1)	0.8 (0.5–1.3)
5	2.4* (2.0–2.9)	2.9* (2.1–4.0)	1.1 (0.8–1.6)	0.8 (0.5–1.4)	0.9 (0.5–1.6)
6	2.8* (2.0–3.8)	4.3* (2.8–6.5)	1.2 (0.7–2.0)	0.7 (0.3–1.7)	1.8 (0.8–4.0)
7	3.8* (2.2–6.6)	3.1* (1.8–5.3)	1.7 (0.9–3.2)	0.7 (0.3–1.8)	
8+	2.7* (1.4–5.2)				
**χ^2^**	269.7*	121.8*	4.2	10.4	4.2

*Significant at the .05 level, two-sided test.

1 Assessed in Part II sample due to having Part II controls. Some countries were missing part of the trauma variables and were coded “No” for those variables: Combat, Exposure to War, Refugee were all coded “No” for India and Brazil, and Natural Disaster also coded “No” for Brazil. For Israel, the entire sample is coded “Yes” for exposure to war with the age of onset set to the age they moved to Israel. Controls for all models included person-year, country, demographic factors (age, sex, time-varying education, time-varying marriage), interactions between life course (3 dichotomous dummies representing early, middle, and later years in the person's life) and demographic variables, parent psychopathology, and childhood adversities (additional details available upon request).

2 For number of events, the last odd ratio represents the odd of the number or more. For example, for the attempt among total sample, 7 events represent 7 or more events (i.e., 7+ events).

### Multivariate associations between type and number of traumatic events and suicidal behavior

Next we examined an interactive multivariate model that included both type and number of traumatic events in the prediction of subsequent first onset of each type of suicidal behavior (Table 5). The ORs for individual traumas in this model can be interpreted as the relative odds of subsequent suicidal behavior among respondents with a history of one and only one traumatic event versus those with no events (and so are somewhat higher than in Table 3). Similar to the additive multivariate model described above, most types of traumatic events are associated with subsequent suicide ideation and attempts; however, none are associated with a significant increase in the odds of transitioning from ideation to plans or attempt. In this more elaborate model that includes type and number of traumatic events, the ORs for number of events are lower than 1.0 in the prediction of suicide ideation and attempt, indicating the existence of sub-additive effects. That is, as the number of traumatic events increases, the relative odds of suicide ideation and attempt increase at a decreasing rate. In other words, as a person experiences more and more traumatic events, the impact of each additional event lessens in magnitude. These sub-additive effects are not observed consistently in the prediction of suicide plan and attempt among those with suicide ideation. A similar pattern of findings holds in high-, middle-, and low-income countries (data available upon request).

**Table 5:** Multivariate model for associations between type and number of traumatic events and suicidal behavior^1^.

	Among Total Sample		Plan among ideators	Attempt among ideators with a plan	Attempt among ideators without a plan
	Ideation	Attempt			
Type of Traumatic Events	OR(95% CI)	OR(95% CI)	OR(95% CI)	OR(95% CI)	OR(95% CI)
Disasters/Accidents					
All Man Made Disasters	1.4* (1.2–1.7)	1.4* (1.1–1.8)	1.2 (0.9–1.6)	0.8 (0.5–1.2)	0.9 (0.6–1.3)
Natural Disaster	1.2 (1.0–1.4)	1.4* (1.1–1.9)	1.3 (1.0–1.7)	1.1 (0.7–1.6)	1.2 (0.8–1.7)
Accident	1.6* (1.4–1.8)	1.6* (1.2–2.0)	1.1 (0.8–1.4)	0.6* (0.4–1.0)	0.7 (0.5–1.1)
War/Combat/Refugee Experiences					
Exposure to War	1.1 (1.0–1.3)	1.5* (1.2–1.9)	1.1 (0.8–1.5)	1.5 (0.9–2.5)	0.5* (0.3–1.0)
Combat	1.3* (1.0–1.8)	1.0 (0.7–1.4)	1.3 (0.8–2.0)	1.0 (0.4–2.2)	0.4* (0.2–0.9)
Refugee	1.3 (0.9–1.7)	1.3 (0.8–2.1)	0.9 (0.5–1.7)	1.2 (0.6–2.7)	1.3 (0.5–3.3)
Sexual/Interpersonal Violence					
Sexual Violence	2.3* (2.0–2.7)	2.9* (2.3–3.6)	1.1 (0.8–1.4)	0.8 (0.6–1.2)	1.3 (0.9–2.0)
Interpersonal Violence	1.9* (1.6–2.1)	2.0* (1.6–2.4)	1.1 (0.8–1.4)	0.6* (0.4–1.0)	0.9 (0.7–1.4)
Witness/Perpetrator Violence					
Witness Violence	1.4* (1.2–1.6)	1.5* (1.2–1.8)	1.2 (0.9–1.6)	0.9 (0.6–1.3)	1.1 (0.7–1.6)
Perpetrator Violence	1.6* (1.3–2.0)	1.7* (1.2–2.3)	1.2 (0.8–1.7)	0.6 (0.3–1.1)	1.0 (0.5–2.1)
Loss/Trauma					
Death of Loved One	1.3* (1.1–1.4)	1.2* (1.0–1.5)	0.9 (0.7–1.1)	0.8 (0.6–1.1)	0.7* (0.5–1.0)
Trauma to Loved one	1.3* (1.1–1.6)	1.5* (1.1–1.8)	0.8 (0.6–1.1)	0.9 (0.6–1.3)	0.8 (0.6–1.2)
All Others	1.7* (1.4–1.9)	1.8* (1.4–2.2)	1.0 (0.8–1.3)	0.8 (0.6–1.3)	0.9 (0.6–1.3)
13 df group significance test for 13 types	239.5*	149.3*	20.7	25.2*	23.8*
12 df significance test for difference between types	157.4*	93.9*	20.1	22.7*	20.6

Number of Traumatic Events 2					
2 events	0.9 (0.7–1.0)	0.8 (0.6–1.0)	1.0 (0.8–1.4)	1.3 (0.8–2.0)	1.3 (0.8–2.1)
3 events	0.6* (0.5–0.8)	0.5* (0.4–0.8)	0.9 (0.6–1.4)	1.1 (0.5–2.4)	1.3 (0.7–2.7)
4 events	0.4* (0.3–0.6)	0.4* (0.2–0.6)	0.8 (0.4–1.5)	1.6 (0.6–4.4)	1.4 (0.5–3.7)
5 events	0.3* (0.2–0.5)	0.3* (0.2–0.6)	0.8 (0.4–1.9)	2.4 (0.7–8.9)	1.8 (0.5–6.7)
6 events	0.3* (0.2–0.5)	0.3* (0.1–0.7)	0.8 (0.3–2.4)	2.7 (0.5–16.3)	3.9 (0.6–25.1)
7 events	0.3* (0.1–0.6)	0.1* (0.0–0.4)	0.9 (0.2–3.7)	3.3 (0.4–29.2)	
8+ events	0.1* (0.0–0.3)				
**χ^2^**	45.5*	22.6*	2.7	5.8	3.6

*Significant at the .05 level, two-sided test.

1 Assessed in Part II sample due to having Part II controls. Some countries were missing part of the trauma variables and were coded “No” for those variables: Combat, Exposure to War, Refugee were all coded “No” for India and Brazil, and Natural Disaster was also coded “No” for Brazil. For Israel, the entire sample is coded “Yes” for Exposure to War with the age of onset set to the age they moved to Israel. Controls for all models included person-year, country, demographic factors (age, sex, time-varying education, time-varying marriage), interactions between life course (3 dichotomous dummies representing early, middle, and later years in the person's life) and demographic variables, parent psychopathology, and childhood adversities (additional details available upon request).

2 For number of events, the last odd ratio represents the odd of the number or more. For example, for the attempt among total sample, 7 events represent 7 or more events (i.e., 7+ events).

Next we tested whether the associations between traumatic events and suicidal behavior are mediated by the presence of mental disorders. Re-estimation of the above models after adjusting for the presence of Axis I mental disorders revealed that the associations between traumatic events and suicidal behavior were largely unchanged. Specifically, the ORs for suicide ideation changed from 1.1–2.3 (CI: 1.0–2.7) in the first model, to 1.1–2.0 (CI: 1.0–2.3) in the adjusted model, whereas the ORs for suicide attempt changed from 1.0–2.9 (CI: 0.7–3.6) to 0.9–2.3 (CI: 0.6–2.8) (detailed results available upon request).

### Persistence of suicidal behavior

Results from the backward recurrence analyses indicate that no specific traumatic events are associated with the persistence of suicide ideation or suicide attempts in the bivariate models (Table 6). However, having experienced one traumatic event is associated with persistence of suicide ideation and attempts. In the multivariate model, several types of traumatic events are predictive of the persistence of suicidal behavior, with exposure to accidents and to sexual violence predicting persistence of both suicide ideation and suicide attempt. These associations are invariably due to traumatic events predicting the persistence of suicide ideation rather than attempts per se (data available upon request). This pattern of findings holds true across high-, middle-, and low-income countries (data available upon request).

**Table 6:** Association between traumatic events and persistence of suicidal behavior^1^.

	Bivariate^2^		Multivariate^3^	
	Ideation	Attempt among ideators	Ideation	Attempt among ideators
Type of Traumatic Events	OR(95% CI)	OR(95% CI)	OR(95% CI)	OR(95% CI)
Disasters/Accidents				
All man made disasters	0.9 (0.8–1.2)	1.2 (0.8–1.8)	1.2 (0.9–1.5)	1.7* (1.0–2.8)
Natural Disaster	0.9 (0.8–1.1)	0.7 (0.4–1.1)	1.1 (0.8–1.4)	0.9 (0.5–1.6)
Accident	1.0 (0.9–1.2)	1.2 (0.8–1.7)	1.3* (1.0–1.6)	1.6* (1.0–2.6)
War/Combat/Refugee Experiences				
Exposure to war	0.9 (0.7–1.2)	1.2 (0.7–2.0)	1.1 (0.9–1.5)	1.7* (1.0–2.9)
Combat	0.8 (0.5–1.3)	1.1 (0.5–2.6)	1.1 (0.7–1.9)	1.6 (0.6–4.5)
Refugee	1.4 (0.9–2.2)	1.6 (0.7–3.6)	1.7* (1.0–2.9)	2.4 (1.0–6.2)
Sexual/Interpersonal Violence				
Sexual Violence	1.1 (1.0–1.3)	1.2 (0.9–1.6)	1.3* (1.1–1.6)	1.6* (1.1–2.3)
Interpersonal violence	1.0 (0.9–1.2)	0.9 (0.5–1.3)	1.2 (1.0–1.4)	1.1 (0.7–1.8)
Witness/Perpetrator Violence				
Witness violence	0.9 (0.8–1.1)	1.1 (0.8–1.5)	1.1 (0.9–1.4)	1.4 (0.9–2.1)
Perpetrator violence	0.8 (0.6–1.1)	0.9 (0.5–1.8)	1.0 (0.7–1.5)	1.2 (0.6–2.5)
Loss/Trauma				
Death of loved one	1.0 (0.9–1.1)	1.3 (1.0–1.7)	1.2 (1.0–1.4)	1.7* (1.2–2.4)
Trauma to loved one	0.9 (0.7–1.0)	1.1 (0.8–1.6)	1.1 (0.8–1.3)	1.5 (1.0–2.3)
All others	1.0 (0.9–1.2)	1.1 (0.8–1.4)	1.2* (1.0–1.5)	1.5 (1.0–2.1)
13 df group significance test for 13 types			16.2	20.0
12 df significance test for difference between types			9.3	10.0

Number of Traumatic Events 4				
1 event	1.2* (1.0–1.4)	1.4* (1.1–1.9)		
2 events	1.0 (0.9–1.2)	1.2 (0.9–1.7)	0.7* (0.6–0.9)	0.6* (0.4–1.0)
3 events	1.1 (0.9–1.4)	1.5* (1.0–2.4)	0.7* (0.5–1.0)	0.5 (0.2–1.1)
4 events	0.8 (0.6–1.1)	1.2 (0.7–2.1)	0.4* (0.2–0.7)	0.3* (0.1–0.7)
5 events	1.0 (0.6–1.5)	1.0 (0.5–1.9)	0.4* (0.2–0.9)	0.1* (0.0–0.5)
6 events	0.8 (0.4–1.4)	1.2 (0.5–2.8)	0.3* (0.1–0.7)	0.1* (0.0–0.6)
7+ events	1.0 (0.6–1.9)		0.3 (0.1–1.0)	
**χ^2^**	12.9	9.3	11.6	10.1

*Significant at the 0.05 level, 2-sided test.

1 Assessed in Part II sample due to having Part II controls. Countries include: Belgium, Brazil, Bulgaria, Colombia, France, Germany, India, Israel, Italy, Japan, Lebanon, Mexico, Netherlands, New Zealand, Nigeria, People's Republic of China, Romania, South Africa, Spain, Ukraine, United States. Results are based on discrete time survival model with country differences, a set of age-related variables (i.e., age, onset and time since onset), sociodemographic variables (including sex, educational attainment and marriage), parent psychopathology, and childhood adversity as a control (additional details available upon request).

2 Each row represents a separate bivariate model; some models do not include data from all countries if the country is missing the variable. India and Brazil were dropped in the bivariate model for Combat, Exposure to War and Refugees; and Brazil was dropped in the bivariate model for Natural Disaster. For Israel, the entire sample is coded “Yes” for Exposure to War with the age of onset set to the age they moved to Israel.

3 Some countries were missing part of the trauma variables and were coded “No” for those variables in the multivariate models: Combat, Exposure to War and Refugee were all coded “No” for India and Brazil; and Natural Disaster was also coded “No” for Brazil. For Israel, the entire sample is coded “Yes” for Exposure to War with the age of onset set to the age they moved to Israel.

4 For number of events, the last odd ratio represents the odd of the number or more. For example, for the attempt among ideators, 6 events represent 6 or more events (i.e., 6+ events).

### Interaction of traumatic events and PTSD

As noted earlier, it has been suggested that the association between traumatic events and suicidal behavior is seen primarily in the context of PTSD [17]. Table 7 shows the interactions between traumatic events and PTSD in predicting suicide ideation and suicide attempt. The relative lack of significant findings suggests that the associations between traumatic events and suicidal behavior do not occur only in the presence of PTSD.

**Table 7:** Suicidal behavior assessed with interactions between DSM-IV PTSD and individual traumatic events^1^.

	Among Total Sample		Plan among ideators	Attempt among ideators with a plan	Attempt among ideators without a plan
	Ideation	Attempt			
Type of Traumatic Events	OR(95% CI)	OR(95% CI)	OR(95% CI)	OR(95% CI)	OR(95% CI)
Disasters/Accidents					
All Man Made Disasters	1.1 (0.6–1.8)	0.6 (0.3–1.2)	0.7 (0.4–1.4)	0.9 (0.2–3.4)	0.3 (0.1–1.3)
Natural Disaster	1.0 (0.6–1.8)	0.9 (0.5–1.8)	0.7 (0.4–1.4)	1.1 (0.4–2.6)	0.5 (0.1–1.7)
Accident	1.1 (0.8–1.7)	0.8 (0.5–1.3)	1.3 (0.8–2.1)	1.1 (0.6–2.3)	0.4 (0.1–1.0)
War/Combat/Refugee Experiences					
Exposure to War	1.6 (0.8–3.1)	1.8 (0.9–3.7)	1.8 (0.8–4.0)	1.0 (0.3–3.8)	2.8 (0.4–18.2)
Combat	1.6 (0.5–4.8)	0.6 (0.2–1.9)	0.6 (0.2–1.5)	0.6 (0.1–4.7)	0.0* (0.0–0.0)
Refugee	0.6 (0.3–1.4)	1.0 (0.4–2.9)	1.1 (0.3–3.5)	0.4 (0.1–2.6)	13.0 (0.6–295.2)
Sexual/Interpersonal Violence					
Sexual Violence	0.9 (0.6–1.3)	1.1 (0.7–1.7)	0.6* (0.3–0.9)	0.8 (0.3–1.9)	0.9 (0.4–2.0)
Interpersonal Violence	0.8 (0.5–1.1)	1.1 (0.7–1.6)	1.1 (0.7–1.7)	0.7 (0.3–1.5)	1.4 (0.7–3.1)
Witness/Perpetrator Violence					
Witness Violence	1.3 (0.9–1.9)	1.3 (0.8–2.1)	0.8 (0.5–1.4)	1.7 (0.8–3.4)	0.9 (0.3–2.6)
Perpetrator Violence	0.4* (0.2–0.9)	1.2 (0.6–2.3)	1.9 (0.7–4.7)	1.4 (0.4–5.0)	2.8 (0.5–14.4)
Loss/Trauma^3^					
Death of Loved One	0.8 (0.6–1.0)	0.8 (0.6–1.2)	0.8 (0.5–1.3)	1.1 (0.6–1.9)	0.9 (0.4–1.8)
Trauma to Loved one	0.9 (0.6–1.2)	0.7 (0.5–1.1)	0.6* (0.4–1.0)	0.8 (0.4–1.6)	2.1 (0.9–5.1)
All Others	0.6* (0.4–0.9)	0.7 (0.5–1.2)	1.0 (0.6–1.6)	1.3 (0.6–2.8)	0.7 (0.3–1.6)
13 df group interaction test	25.2*	18.9	21.1	5.5	224.0*

*Significant at the .05 level, two-sided test.

1 Multiviate models included interaction terms between DSM-IV PTSD and each trauma event. Only interaction terms are shown in the table, while the main effects are still controlled for. Assessed in Part II sample due to having Part II controls. Some countries were missing part of the trauma variables and were coded “No” for those variables: Combat, Exposure to War, Refugee were all coded “No” for India and Brazil, and Natural Disaster also coded “No” for Brazil. For Israel, the entire sample is coded “Yes” for exposure to war with the age of onset set to the age they moved to Israel. Controls for all models included person-year, country, demographic factors (age, sex, time-varying education, time-varying marriage), interactions between life course (3 dichotomous dummies representing early, middle, and later years in the person's life) and demographic variables, parent psychopathology, and childhood adversities (additional details available upon request).

### Population attributable risk proportions

Finally, we calculated PARPS to examine the population-level effects of traumatic events on suicidal behavior. Results revealed that, assuming a causal relation between traumatic events and suicidal behavior, the elimination of all traumatic events would lead to a 15.4% reduction in suicide ideation and a 22.1% reduction in suicide attempts (Table 8). Consistent with prior analyses, these effects were due primarily to the association between traumatic events and suicide ideation, as PARPs for plans and attempts among ideators were approximately zero (−1.0% to 0.3%).

**Table 8:** Total (all countries combined) PARP of trauma among suicidality^1^.

	Among Total Sample		Plan among ideators	Attempt among ideators with a plan	Attempt among ideators without a plan
	Ideation	Attempt			
Type of Traumatic Events	PARP	PARP	PARP	PARP	PARP
Disasters/Accidents					
All Man Made Disasters	0.26%	0.24%	0.08%	−0.02%	−0.01%
Natural Disaster	−0.04%	0.52%	0.20%	0.07%	0.31%
Accident	2.11%	2.06%	0.03%	−0.17%	−0.11%
War/Combat/Refugee Experiences					
Exposure to War	−0.62%	1.31%	0.10%	0.46%	−1.03%
Combat	−0.09%	−0.49%	0.05%	0.02%	−0.07%
Refugee	−0.06%	0.02%	−0.03%	0.02%	0.06%
Sexual/Interpersonal Violence					
Sexual Violence	4.18%	7.82%	0.02%	−0.03%	0.17%
Interpersonal Violence	4.80%	5.88%	0.06%	−0.19%	0.07%
Witness/Perpetrator Violence					
Witness Violence	1.58%	1.90%	0.25%	−0.02%	0.19%
Perpetrator Violence	0.27%	0.45%	0.02%	−0.03%	0.02%
Loss/Trauma					
Death of Loved One	0.92%	−0.34%	−0.34%	−0.11%	−0.32%
Trauma to Loved one	0.36%	0.88%	−0.16%	0.00%	−0.02%
All Others	1.65%	2.53%	−0.01%	−0.01%	−0.01%
All Traumatic Events	15.41%	22.06%	0.33%	−0.03%	−0.93%

1 Each row represent separate models calculating PARP by curing each trauma individually and all combined (final row). Controls are the same as Table 2, 3, 4, 5 for each column.

## Discussion

Several limitations of the analyses should be emphasized. First, not all potential traumas are listed in detail in the PTSD module; the residual “other trauma” category may include important traumas such as human rights violations [32]. Similarly, the severity and duration of individual traumas are not assessed. Although we obtained detailed data on trauma exposure, the characteristics of trauma may be important, for example, in predicting the transition from suicide ideation to suicide attempt. Second, data from various parts of the globe may differ in important respects; there were different response rates in different countries, and not all samples are nationally representative. Although we controlled for differential response using post-stratification adjustments, response rates may have been related to trauma exposure or suicidal behavior, limiting the generality of the estimates. Third, it is important to emphasize that assessment of both traumatic events and suicidal behavior is based on retrospective self-report. Although significant attention was paid to questionnaire methodology to maximize respondents' recall and to minimize reporting differences, the data are subject to biases at the level of the individual (e.g., mood-congruent recall bias), and of the cultural context (e.g., different cultural contexts may have influenced responses to questions about trauma and suicide in different ways across the surveys) [33]–[36].

Nevertheless, these data provide a more fine-grained analysis of the relationship between traumatic events and suicidal behavior than has previously been possible, and in doing so extend previous data from community and clinical studies [14], [17], [37]–[39]. Our main findings were that: (1) in multivariate models there is a particularly strong association between sexual and interpersonal violence and suicide ideation/attempt; (2) there is a dose-response relationship between the number of traumatic events experienced and the subsequent odds of suicide ideation/attempt, but the effects are subadditive with a decay in the strength of the association with more events; (3) although specific traumatic events are useful in predicting suicide ideation, they are generally less useful in predicting the progression from suicide ideation to attempt; and (4) the general pattern of findings holds true across high-, middle-, and low-income countries, regardless of the presence of PTSD, and are not mediated by the presence of mental disorders.

Previous work has emphasized the relationship between exposure to sexual and interpersonal violence and suicidality [16], [40]–[43]. A range of different mechanisms may account for the specificity of these associations. Disruptions in interpersonal and social bonds (both current and future), for example, may play a key role in precipitating suicide in those who are most vulnerable. Exposure to sexual and interpersonal violence are associated (as are other traumas) with psychiatric disorders such as depression and PTSD, but also (perhaps more specifically than certain other traumas) with increased impulsivity [44], which may play a key role in stress-diathesis models of suicide [11], [14], [16], [41], [45]. The finding that many other traumas are associated with suicidal behavior in bivariate but not multivariate models underscores the complexity of the associations between traumatic events and suicidal behavior. This pattern of findings suggests that some types of traumatic events may be associated with suicidal behavior only because they co-occur with other events that are themselves uniquely associated with suicidal outcomes. For instance, being the perpetrator of violence against others is associated with a subsequent suicide attempt in the bivariate, but not multivariate, analysis, and this may be because the association between these two variables is explained by witnessing violence (even when one is the perpetrator). An alternative hypothesis is that the associations between traumatic events and suicidal behavior are explained by some element common to all such events so that when all are included in a model simultaneously, the unique contribution of each type of event is substantially diminished. However, the fact that most events remained significantly associated with suicide attempt in the multivariate model suggests that this cannot fully explain the observed associations.

The data here are also useful in demonstrating that although more traumatic events are associated with increased suicidal behavior, this influence increases at a decreasing rate—perhaps due in part to habituation. These findings are consistent with a stress-diathesis theory of suicide in which trauma initiates a stress response with biological and psychological consequences (e.g., increased distress or hopelessness) and in which multiple traumas increase the strength of the stress response, but with other factors playing a role in predisposing one to suicide ideation and attempt. We found that certain kinds of trauma, such as accidents and sexual violence, are predictive of the persistence of suicide ideation/attempts; stress-diathesis models of suicidal behavior need further elaboration in order to address the complexities of severity and timing of both risk factors and suicide outcomes.

The data here also indicate that the association between traumatic events and suicide attempt is largely due to traumatic events predicting suicide ideation rather than to the progression from suicide ideation to attempt. Nevertheless, in the cross-national sample, among those with suicide ideation, natural disaster is associated with suicide plan, exposure to war is associated with planned attempt, and sexual violence is associated with unplanned attempt. These data are to some extent consistent with current knowledge of the different kinds of psychopathology that follow different traumatic events; exposure to natural disasters and war may lead to phenomena such as survivor guilt and planned suicide, while exposure to sexual violence may be associated with a range of more impulsive psychopathology [44], [46]. On balance, this pattern was not observed consistently across high-, middle-, and low-income countries, suggesting that these particular associations should be interpreted with some caution until they are shown to replicate across individual countries and/or studies.

Despite this lack of consistency in the risk factors for transitions from suicide ideation to suicide plan and attempt, it is notable that the observed risk factors for suicide ideation and attempt more generally were quite similar across high-, middle-, and low-income countries. This is consistent with growing research on the risk factors for suicidal behavior, many of which cut across a range of different contexts [11]. For example, while prevalence of both psychiatric disorders and suicidal behavior differs across countries, the associations between disorders and suicidal behavior are quite consistent cross-nationally [11]. The consistent pattern of results across different regions of the globe provides significant support for the validity of the associations documented here, despite the limitations noted earlier.

In contrast to the previous work by Wilcox and colleagues [17], we found that the relationships between traumatic events and suicidal behavior held irrespective of whether or not PTSD was present. That study was, however, limited to a young sample of urban African American adults. The findings here are consistent with a view that the mechanisms underlying the relationship between trauma exposure and suicidality are multiple, and may not be explicable on the basis of any single psychiatric entity, or even by psychiatric disorders more generally. Further work is needed to explore in detail the interactions between childhood-onset adversities, adult-onset traumas, and different Axis I and II disorders in the prediction of suicidal behavior [47].

The findings here have potentially important implications not only for mental health policy but also for clinical assessment and intervention. From a policy perspective, there is increasing awareness of violence and other traumas as a major public health problem [48], requiring robust multi-sectoral intervention across the globe. Prevention of traumas, particularly sexual and interpersonal violence, may ultimately result in a significantly reduced burden of psychiatric disorder, including suicide ideation and attempts. In the clinic, it would seem crucial to routinely assess patients for exposure to trauma, including multiple traumas, particularly when there is evidence of psychopathology, including suicide ideation or suicide attempts. Although the results of this study suggest that completely eliminating traumatic events would lead to at most a 22.1% reduction in suicide attempts, future research should examine whether clinical and policy interventions aimed at decreasing the occurrence and impact of traumatic events are effective in decreasing suicidal behavior.
